# A Nasal Resistance Measurement System Based on Multi-Sensor Fusion of Pressure and Flow

**DOI:** 10.3390/mi16080886

**Published:** 2025-07-29

**Authors:** Xiaoqin Lian, Guochun Ma, Chao Gao, Chunquan Liu, Yelan Wu, Wenyang Guan

**Affiliations:** 1 School of Computer and Artificial Intelligence, Beijing Technology and Business University, Beijing 100048, China; lianxq@th.btbu.edu.cn (X.L.); 2230601019@st.btbu.edu.cn (G.M.); 2330602060@st.btbu.edu.cn (C.L.); wuyel@th.btbu.edu.cn (Y.W.); guanwenyang@btbu.edu.cn (W.G.); 2 Key Laboratory of Industrial Internet and Big Data, China National Light Industry, Beijing Technology and Business University, Beijing 100048, China

**Keywords:** nasal resistance, multi-sensor fusion, respiratory monitoring, auxiliary diagnosis

## Abstract

Nasal obstruction is a common symptom of nasal conditions, with nasal resistance being a crucial physiological indicator for assessing severity. However, traditional rhinomanometry faces challenges with interference, limited automation, and unstable measurement results. To address these issues, this research designed a nasal resistance measurement system based on multi-sensor fusion of pressure and flow. The system comprises lower computer hardware for acquiring raw pressure–flow signals in the nasal cavity and upper computer software for segmenting and filtering effective respiratory cycles and calculating various nasal resistance indicators. Meanwhile, the system’s anti-interference capability was assessed using recall, precision, and accuracy rates for respiratory cycle recognition, while stability was evaluated by analyzing the standard deviation of nasal resistance indicators. The experimental results demonstrate that the system achieves recall and precision rates of 99% and 86%, respectively, for the recognition of effective respiratory cycles. Additionally, under the three common interference scenarios of saturated or weak breaths, breaths when not worn properly, and multiple breaths, the system can achieve a maximum accuracy of 96.30% in identifying ineffective respiratory cycles. Furthermore, compared to the measurement without filtering for effective respiratory cycles, the system reduces the median within-group standard deviation across four types of nasal resistance measurements by 5 to 18 times. In conclusion, the nasal resistance measurement system developed in this research demonstrates strong anti-interference capabilities, significantly enhances the automation of the measurement process and the stability of the measurement results, and offers robust technical support for the auxiliary diagnosis of related nasal conditions.

## 1. Introduction

The nasal cavity is the primary passage of the respiratory system, and the changes in its resistance can reflect the health status and physiological function of the respiratory system. With the development of measurement technology, nasal resistance measurement has become an important method for nasal cavity function assessment [1,2,3,4]. It is widely used in the auxiliary diagnosis and treatment planning of nasal-related diseases such as allergic rhinitis [5,6] and sinusitis [7,8], as well as in the monitoring of treatment efficacy [9]. Additionally, nasal resistance measurement can be used to evaluate the severity and treatment effectiveness of obstructive sleep apnea (OSA) [10,11]. Furthermore, this technology is utilized in sports medicine to monitor athletes’ nasal function, helping to optimize breathing efficiency [12,13]. Therefore, accurate and real-time measurement of nasal resistance is of significant importance. Nasal resistance measurement methods can be categorized into subjective and objective methods [14,15]. Subjective methods rely on patient self-reports or clinician evaluations. These methods include subjective testing by clinicians, such as nasendoscopy [16], and subjective measures by patients, such as the Nasal Obstruction Symptom Evaluation (NOSE) scale [17]. In contrast, objective methods utilize diagnostic equipment and employ techniques such as laboratory tests and imaging analysis to obtain quantifiable data for evaluating the patient’s health condition. Objective methods can be further divided into measurements of nasal cavity structure and function. Compared to subjective methods, objective methods provide more precise diagnostic information for clinicians, facilitating more accurate treatment decisions.

Nasal cavity structural measurement is a crucial method for investigating the anatomical structure of the nasal cavity, confirming pathological diagnoses, and monitoring disease recurrence [15]. Methods such as Computed Tomography (CT) [15,18], Magnetic Resonance Imaging (MRI) [15], and acoustic testing [19,20] are frequently employed for nasal cavity structural measurement. These methods utilize image processing and acoustic reflection technologies to reconstruct the nasal cavity structure, analyzing the volume occupied by internal organs or tissues and the relationship between the cross-sectional area and the depth of the nasal cavity. This information provides a foundation for diagnosis and surgical planning. However, these methods are limited in their ability to describe the fluid dynamics within the nasal cavity during respiration. Additionally, the dynamic changes of the nasal cavity during breathing are not considered [15,21], resulting in a weak correlation with subjective nasal obstruction experiences. Furthermore, structural measurement typically requires expensive equipment and may carry risks of radiation exposure [22]. Computational Fluid Dynamics (CFD) is a multidisciplinary approach based on CT or MRI measurement results that integrates fluid mechanics, mathematics, and computer science to simulate various parameters in the nasal cavity, such as airflow, pressure distribution, and heat and moisture exchange. However, it demands substantial computational power and is often associated with long calculation times [21,23]. Additionally, this method easily neglects the elastic deformation of the nasal cavity walls during breathing, which can affect the accuracy and reliability of the results [24]. Therefore, how to improve the reliability and accuracy of measurements while reducing costs is still a topic of widespread research in order to facilitate its future clinical applications.

Unlike structural measurement, nasal function measurement focuses on the breathing process, allowing dynamic recording of pressure and flow changes within the nasal cavity during respiration; thus, the nasal resistance can be quantified. Flow sensors are frequently used to detect flow variations in the nasal cavity during breathing. Youlten et al. [25] placed a Mini Wright peak flow meter [26] at the end of a sealed oronasal mask to collect real-time flow data and record the peak nasal flow during forced nasal inspiration [27,28]. This method evaluates nasal resistance using peak flow, offering a quick, reliable, and repeatable assessment. Moreover, the equipment is affordable, portable, and does not require operation under the guidance of professional testing personnel [15]. However, relying solely on a single flow sensor can introduce interference from lung function, which may affect the physician’s assessment of nasal obstruction severity [15,29,30].

To address the limitations of using a single sensor, Clement et al. [31] utilized both flow and pressure sensors to collect flow and pressure changes within the nasal cavity during respiration, allowing for the calculation of resistance at specific pressure levels. Building on this method, Vogt and Hoffrichter divided the collected respiratory data into four phases and applied statistical analysis methods to comprehensively evaluate nasal resistance by analyzing the pressure and flow across these phases [32,33]. This method offers high sensitivity and shows a strong correlation with patients’ subjective nasal obstruction experiences [22]. Significantly, this method is an internationally standardized method for assessing nasal obstruction and is recommended as the gold standard tool for measuring nasal resistance [34,35]. Despite being the best method for measuring nasal resistance, this method has certain requirements for the indoor environment during testing and necessitates patient cooperation under the guidance of professional personnel [15,22]. For example, the patient needs to maintain a stable breathing pattern and wear a breathing mask properly [36,37]. Additionally, environmental noise is easily introduced during actual testing scenarios. These issues can impact the quality of respiratory data collection, thereby affecting the accurate calculation of nasal resistance values. Ultimately, this makes the method prone to errors and results in poor test–retest reliability [22]. Moreover, there is a lack of comprehensive research on common types of abnormal respiratory data during testing, with no established classification paradigms for abnormal respiratory data, and the measurement system lacks effective preprocessing methods for handling abnormal data. Therefore, doctors or testing personnel need to subjectively filter the collected respiratory data based on their experience and manually eliminate abnormal respiratory data after the test to ensure the accuracy and reliability of the method. Recently, the widespread adoption of time–frequency-domain analysis techniques has enabled the development of more reliable and intelligent measurement systems across various fields, including medical testing [38,39], civil engineering [40], manufacturing [41,42], battery management [43], and industrial testing [44,45,46,47]. These technological advancements present potential solutions to the current limitations of nasal resistance measurement systems, such as poor anti-interference capabilities, limited automation, and unstable measurement results.

Time–frequency-domain analysis is a powerful tool for signal analysis, particularly suitable for processing non-stationary signals. This technology allows signals to be analyzed simultaneously in both time and frequency dimensions, revealing the time and frequency characteristics of the signals. In the medical testing field, various physiological signals, such as Electroencephalogram (EEG) [38,48], voice signals [49], and heartbeat signals [50,51], can be identified and evaluated using time–frequency-domain analysis. This technique can replace manual experience to complete signal filtering and recognition, thereby optimizing the diagnostic workflow and enhancing the efficiency of disease diagnosis.

In this research, a time–frequency-domain analysis method for respiratory pressure–flow signals was proposed, which refines the preprocessing method. Based on this method, a multi-sensor fusion nasal resistance measurement system was designed to address the limitations of current nasal resistance measurement techniques. This system enhances anti-interference capabilities, improves the level of automation, and increases the stability of measurement results. The main research contents include the following: (1) A nasal pressure–flow signal acquisition device was constructed to collect raw pressure–flow signals during breathing. (2) The characteristics of abnormal respiratory data were analyzed to summarize common paradigms. (3) A preprocessing method was designed to segment the respiratory cycles from the raw pressure–flow signals. Meanwhile, effective respiratory cycles were automatically selected from the respiratory cycles utilizing the time–frequency-domain analysis method. (4) An upper computer platform was developed to calculate various nasal resistance indicators based on the principles of classic rhinomanometry [52], 4-phase rhinomanometry [32], and Broms resistance [53].

## 2. Methods

The core challenge in nasal resistance measurement lies in the acquisition of stable, undisturbed pressure–flow signals that accurately reflect the subject’s respiratory status. Traditional rhinomanometry techniques, although standardized, are highly sensitive to environmental noise, user cooperation, and respiratory irregularities, which often result in signal instability and inaccurate assessment of nasal resistance.

In this study, we propose a novel multi-sensor fusion measurement framework combining pressure and flow signals, coupled with a real-time signal validation mechanism based on time–frequency-domain analysis. Unlike existing systems that rely on retrospective manual filtering, our system performs real-time cycle-level validation and segmentation, allowing only effective respiratory cycles to be used in nasal resistance calculation. This approach enhances automation, improves signal quality, and ensures measurement repeatability. The following sections detail the overall architecture of the system and the methods used for signal processing.

### 2.1. Nasal Resistance Measurement System Architecture

**Figure 1:**
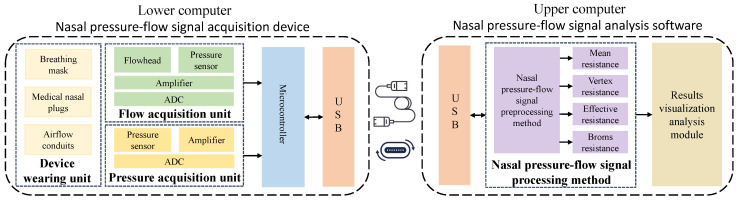
Nasal resistance measurement system architecture diagram.

**Figure 2:**
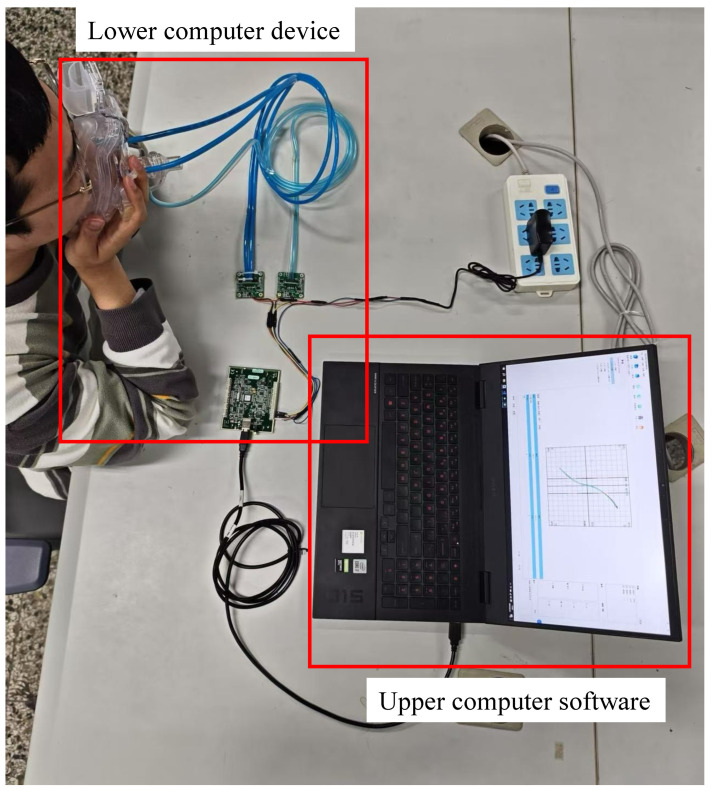
Experimental setup schematic.

In this research, a nasal resistance measurement system was designed based on the study of respiratory pressure–flow signals. The system was primarily composed of two parts: the lower computer nasal pressure–flow signal acquisition device and the upper computer nasal pressure–flow signal analysis software. The architecture of the nasal resistance measurement system is illustrated in Figure 1, and a picture of the experimental setup is shown in Figure 2. The respiratory data used in this study were collected by this system.

The lower computer nasal pressure–flow signal acquisition device consists of a device-wearing unit, a flow acquisition unit, a pressure acquisition unit, a microcontroller, and a USB communication interface. The raw pressure–flow signals during breathing in the nasal cavity are collected by this device. The upper computer nasal pressure–flow signal analysis software consists of a USB communication interface, a nasal pressure–flow signal processing method, and a results visualization analysis module. This software sends acquisition commands to the lower computer device via the USB interface, receives raw pressure–flow signal data from the lower computer device, and calculates various nasal resistance indicators using the nasal pressure–flow signal processing method, thereby visually displaying the pressure–flow curves. Detailed design information of the lower computer of nasal pressure–flow signal acquisition device and the upper computer of nasal pressure–flow signal analysis software is provided in Appendix A.1 and Appendix A.2, respectively. The specific interface layout and functional demonstration of the upper computer software platform are presented in Appendix A.3.

The workflow of the nasal resistance measurement system is as follows: First, an acquisition command is given by the upper computer software. Simultaneously, the testee is required to wear a breathing mask, nasal plugs, and other devices, and is guided to breathe calmly [37]. After the acquisition command is received, the pressure and flow changes generated during breathing are collected by the lower computer device using the flow acquisition unit and the pressure acquisition unit. These signals are amplified and quantified, then sent to the upper computer device via the microcontroller and USB interface for further processing. Subsequently, the raw pressure–flow signal data are received by the upper computer software, and various medical indicators of the testee nasal cavity, such as mean resistance [52], vertex resistance [32], effective resistance [32], and Broms resistance [53], are analyzed using the nasal pressure–flow signal processing method. Then, the breathing process and pressure–flow curves are visually displayed using data visualization methods. Finally, the analysis results are structurally stored.

### 2.2. Nasal Pressure–Flow Signal Processing Methods

**Figure 3:**
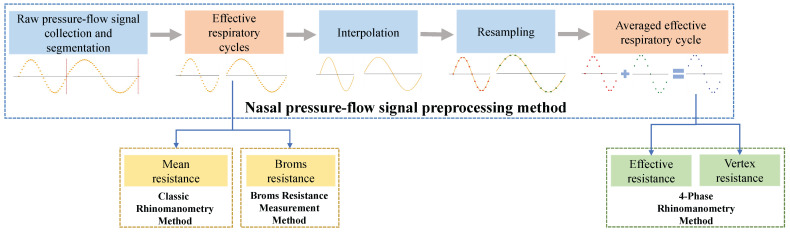
Nasal pressure–flow signal processing methods flow diagram.

The overall architecture of the nasal pressure–flow signal processing method is shown in Figure 3. It mainly includes the nasal pressure–flow signal preprocessing method, the classic rhinomanometry method, the Broms resistance measurement method, and the 4-phase rhinomanometry method. The nasal pressure–flow signal preprocessing method performs segmentation and filtering, interpolation, and resampling of the raw pressure–flow signals. The filtered effective respiratory cycles are used as input data for the classic rhinomanometry method and the Broms resistance measurement method. The interpolated and resampled data can be averaged and used as input for the 4-phase rhinomanometry method [32]. Those methods quantify resistance during breathing from an aerodynamic perspective.

#### 2.2.1. Effective Respiratory Cycles and Ineffective Respiratory Cycles

The collected raw pressure–flow signal is a discrete-time series containing multiple respiratory cycles. These cycles may be subject to varying degrees of interference during the collection process. Therefore, it is necessary to evaluate the quality of these cycles. During nasal resistance measurement, it is essential to use undisturbed data as the source for calculating various nasal resistance indicators to ensure their reliability. In this research, the respiratory cycles within the raw pressure–flow signal are classified into effective and ineffective respiratory cycles based on different scenarios of interference. To improve data quality, a time–frequency analysis method can be applied to the raw pressure–flow signals to identify effective respiratory cycles as well as various types of ineffective respiratory cycles caused by interference. This method is described in detail in the following sections.

**Figure 4:**
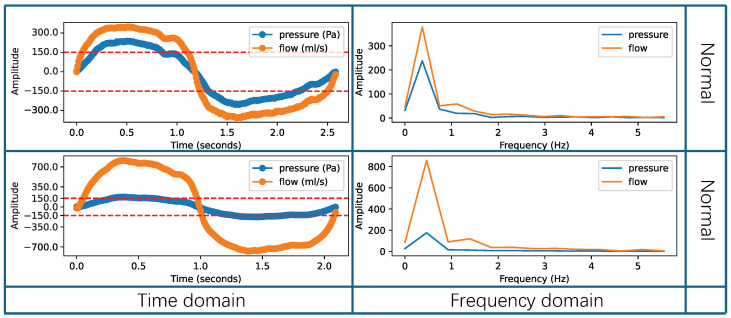
Example of the effective respiratory cycles.

An example of effective respiratory cycles is shown in Figure 4, where the respiratory data are smooth and unaffected by external interferences. The figure analyzes both the time-domain and frequency-domain distributions of the effective respiratory cycle. Each row in the figure represents a single respiratory cycle under normal scenarios, where the respiratory data are not affected by any interference. The orange line represents the flow, and the blue line represents the pressure. The first column of Figure 4 presents the time-domain sequences, with positive intensity parts corresponding to inspiratory phases and negative intensity parts corresponding to expiratory phases. The pressure curve fluctuations range up to ±150 Pa, meeting the requirements for nasal resistance measurement [36,52]. In the frequency domain, statistics show that the adult respiratory frequency is approximately 0.2 Hz to 0.3 Hz [54], while for children over three years old it is approximately 0.3 Hz to 0.5 Hz [55]. The second column in Figure 4 displays the frequency-domain distribution, indicating that the frequency is mainly around 0.3 Hz, consistent with clinical statistics. In summary, when the respiratory cycle’s frequency is primarily around 0.3 Hz and the pressure curve’s fluctuation range reaches ±150 Pa, it can be considered an effective respiratory cycle. These data can be used for calculating nasal resistance indicators.

**Figure 5:**
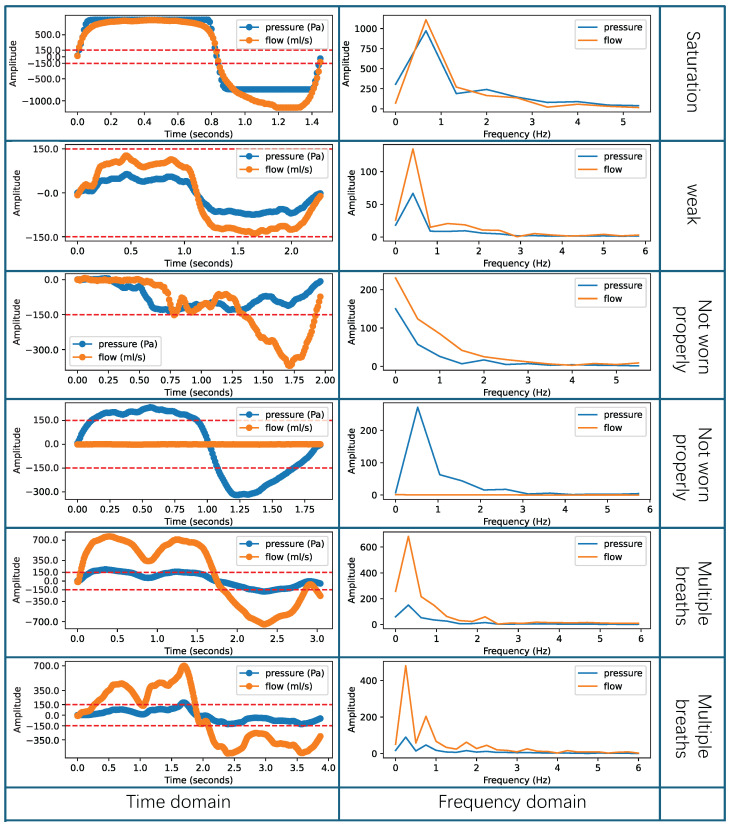
Example of the ineffective respiratory cycles.

However, some respiratory cycles can be influenced by external interference, such as rapid changes in indoor airflow and environmental electronic noise, as well as by human factors, like improperly wearing a breathing device, taking multiple breaths, or interruptions in breathing. These abnormal respiratory data are called ineffective respiratory cycles. This research categorizes the types of ineffective respiratory cycles into three abnormal patterns: saturated or weak breaths, breaths when not worn properly, and multiple breaths. In Figure 5, rows 1–2 contain data for saturated and weak breaths. Saturated breathing results in distorted data because the actual air pressure and flow intensity exceed the maximum intensity detectable by the sensor. On the other hand, during weak breathing, the recorded pressure values do not reach ±150 Pa, which makes it impossible to calculate accurate nasal resistance indicators. In Figure 5, rows 3–4 present data collected when the breathing device was not worn properly. Improper wearing of the breathing device during data collection can lead to missing genuine respiratory data or the inclusion of substantial environmental noise. As shown in Figure 5, this abnormal pattern significantly affects both the time-domain characteristics and frequency-domain distribution of the respiratory cycles. When the breathing device is not properly worn, data from the inspiratory or expiratory phases may be lost in the time domain, resulting in all data intensity values being positive or negative. Moreover, this abnormal pattern may also cause the loss of flow or pressure data, leading to zero intensity values for flow or pressure data in the time domain. In addition to this, in the frequency domain, low-frequency components are likely to be introduced, shifting the overall frequency distribution to the left and deviating from the normal respiratory frequency range. In Figure 5, rows 5–6 display data indicating the occurrence of multiple breaths. Multiple breaths can occur during the inspiratory phase, the expiration phase, or both phases simultaneously. When multiple breaths occur, it significantly affects the peak values of the pressure and flow data in the time domain, resulting in reduced peak intensity. At the same time, in the frequency domain, it introduces high-frequency components, increasing the energy intensity of frequencies above the normal respiratory frequency range. As a result, the presence of these ineffective respiratory cycles increases the measurement error of nasal resistance, rendering the results unsuitable for medical measurement requirements.

#### 2.2.2. Raw Pressure–Flow Signal Collection and Segmentation

The current methods for collecting and segmenting raw pressure–flow signals primarily use zero-crossing detection. After that, doctors or technicians manually select the effective respiratory cycles. To address the time-consuming and labor-intensive nature of manual selection, this research proposed a real-time method for collecting raw pressure–flow signals and automatically segmenting them. This method integrates a segmentation algorithm based on time–frequency-domain analysis. The algorithm can be executed synchronously with data collecting, efficiently segmenting effective respiratory cycles, which reduces the time required for manual secondary selection.

**Figure 6:**
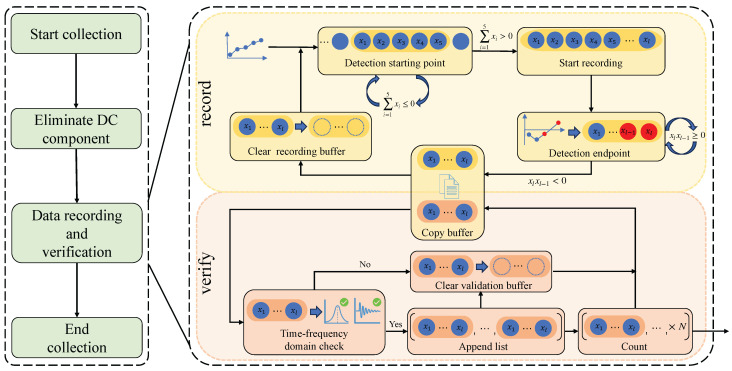
Raw pressure–flow signal collection and segmentation flow diagram.

As shown in Figure 6, the flow diagram illustrates the process of raw pressure–flow signal collection and segmentation. The process mainly consists of four stages: (1) Start collection, (2) Eliminate DC component, (3) Data recording and validation, and (4) End collection. Among these, the data recording and validation stage is the core of the entire process. In this stage, recording and validation threads are created, enabling the synchronous parallel operation of data recording and time–frequency-domain analysis.

The primary purpose of the recording thread is to collect and record respiratory cycle data, consisting of five main steps: Detection Starting Point, Start Recording, Detection Endpoint, Copy Buffer, and Clear Recording Buffer. The recording thread operates at a fixed sampling frequency of 100 Hz, which is substantially higher than the normal respiratory frequency range (0.2 Hz to 0.5 Hz) [54,55]. This satisfies the Nyquist sampling criterion, ensuring that the respiratory signal can be accurately captured and reconstructed for downstream analysis. As illustrated in Figure 6, upon initiation, the thread first executes the Detection Starting Point step. In this step, the program establishes a recording buffer and sequentially stores the collected data in this buffer. It then sums every five consecutive collected data points. If the result of this sum is less than or equal to zero, it indicates that the current data are not part of the inspiratory phase and will not be recorded. Conversely, if the sum is greater than zero, it indicates that the current data belongs to the inspiratory phase. At this time, the Start Recording step is triggered, allowing subsequent data to continue being stored in the recording buffer. As data continues to be collected and stored sequentially, the program proceeds to the Detection Endpoint step. During this step, the program iteratively evaluates the last two elements in the buffer to determine the end of a respiratory cycle. If the product of the last two elements, $x_{\left(l-1\right)}x_{l}$, is greater than zero, it indicates that the current respiratory cycle has not ended. On the other hand, if $x_{\left(l-1\right)}x_{l}$ is less than zero and $x_{\left(l-1\right)}$ is less than zero, it indicates the alternation between inspiratory and expiratory phases, marking the end of the current respiratory cycle. Concurrently, the validation thread is employed to analyze and validate whether or not the recorded respiratory cycle is effective. To efficiently transfer respiratory data between the recording thread and the validation thread, a copying mechanism was employed to connect the buffers within the two threads.

The primary purpose of the validation thread is to filter respiratory cycles and obtain effective respiratory cycles. It mainly includes four steps: Time–Frequency-Domain Check, Append List, Clear Validation Buffer, and Count. As shown in the lower part of Figure 6, upon initiation, the thread initially performs the Time–Frequency-Domain Check step. In this step, the data in the validation buffer are analyzed using the time–frequency-domain analysis algorithm to determine if the frequency range and signal amplitude of the respiratory cycle meet the standards for the effective respiratory cycle. If both the frequency and amplitude standards are met, the respiratory cycle is considered effective. Next, the program executes the Append List step, where each effective respiratory cycle is sequentially stored in the final output list. Finally, the program executes the Count step, counting the number of effective respiratory cycles in the output list. Once the expected number is reached, this list is used as the output of the collection process.

The raw pressure–flow signal collection and segmentation method proposed in this research accomplishes the collection and segmentation in real-time. This method validates each respiratory cycle in parallel based on a multithreading approach. Additionally, by introducing the time–frequency-domain analysis algorithm, the method verifies respiratory cycles, filters out effective ones, and removes ineffective respiratory cycles affected by interference. This method provides a reliable data source for subsequent nasal resistance calculations.

#### 2.2.3. Time–Frequency-Domain Analysis Algorithm

To improve data quality and enhance the reliability of nasal resistance measurement, this research applies time–frequency analysis to nasal pressure–flow signals to determine whether or not each respiratory cycle is effective and to select effective respiratory cycles accordingly. Unlike existing manual selecting methods, the research introduced an innovative algorithm for the analysis of respiratory cycles based on both time-domain and frequency-domain approaches to select effective respiratory cycles. This algorithm facilitates effective respiratory cycle selection by utilizing FFT (Fast Fourier Transform) and VPP (Voltage Peak-to-Peak) thresholds. Initial frequency-domain analysis is conducted using the Fast Fourier Transform to obtain the spectrum of respiratory cycles. Statistical data indicates that normal human respiratory frequency typically falls within the range of approximately 0.2 Hz to 0.5 Hz [54,55]. Considering that nasal resistance measurement includes both healthy individuals and patients, the frequency threshold range was fine-tuned through multiple experiments within and around this range. Specifically, the algorithm determines a Min FFT Threshold (MINFT) and a Max FFT Threshold (MAXFT) to define the acceptable frequency range for effective respiratory cycles. These thresholds were obtained through an iterative optimization process conducted on 280 cases, covering a wide spectrum of effective and ineffective respiratory cycles, including those with varying degrees of nasal obstruction. By evaluating classification performance at different threshold combinations with a step size of 0.01 Hz, the optimal MINFT and MAXFT were determined. This process enables the algorithm to distinguish between effective and ineffective respiratory cycles by analyzing their spectral distributions, thereby supporting accurate and reliable nasal resistance measurements.

On the other hand, the algorithm also performs time-domain analysis by setting a VPP threshold. Since the VPP value of the signal can intuitively reflect its amplitude, it is used as the object of analysis in the time domain. The amplitude of the respiratory cycle is crucial for nasal resistance measurement. The medical committee stipulates that a respiratory pressure of 150 Pa and the corresponding flow data are used as the data source for calculating nasal resistance [36,52]. Thus, if the signal amplitude is too low, some nasal resistance indicators may become unmeasurable. Conversely, excessively high amplitudes can cause the ADC to enter saturation, distorting the signal and impairing measurement accuracy. To address this, four thresholds were established to define the effective VPP ranges: the Minimum and Maximum VPP Thresholds for Pressure signals (MINPVT and MAXPVT), and the Minimum and Maximum VPP Thresholds for Flow signals (MINFVT and MAXFVT). Consistent with the frequency-domain threshold tuning, these thresholds were determined through iterative optimization on the same set of 280 respiratory cycle cases with varying amplitude characteristics. By systematically evaluating classification accuracy over a range of VPP values, the optimal combination of MINPVT, MAXPVT, MINFVT, and MAXFVT was obtained, enabling the algorithm to effectively distinguish between effective and ineffective respiratory cycles in the time domain. In summary, according to Equation (1) the algorithm finally selects effective respiratory cycles ${\left\{x_{i}^{n}\right\}}_{i=1}^{L_{n}}$ using different FFT and VPP thresholds, where *n* represents the group of effective respiratory cycles and $L_{n}$ denotes the length of this cycle.

(1) $${\left\{x_{i}^{n}\right\}}_{i=1}^{L_{n}}\in \mathrm{Effective}\;\mathrm{Respiratory}\;\mathrm{Cycles}\Leftrightarrow \left\{\begin{array}{c} MINFT\leq f_{cycle}\leq MAXFT\\ MINPVT\leq vpp_{cycle}^{p}\leq MAXPVT\\ MINFVT\leq vpp_{cycle}^{f}\leq MAXFVT \end{array}\right.$$

where $f_{cycle}$ is the dominant frequency obtained by FFT, $vpp_{cycle}^{p}$ is the pressure signal’s peak-to-peak value, and $vpp_{cycle}^{f}$ is the flow signal’s peak-to-peak value for the respiratory cycle.

#### 2.2.4. Effective Respiratory Cycle Interpolation and Resampling

When performing nasal resistance measurement based on the 4-phase rhinomanometry method, multiple consecutive tests are often required to obtain more reliable results, followed by averaging the outcomes [56]. Since the duration of effective respiratory cycles varies between tests, direct addition and averaging of these segments are not feasible. To address this issue, this research first employed interpolation technique to convert the discrete effective respiratory cycle sequences into continuous function expressions. Subsequently, a resampling technique was used to sample the continuous functions, generating discrete sequences with consistent lengths. This facilitated the calculation of the average value. Specifically, a discrete effective respiratory cycle of length $L_{n}$, denoted as ${\left\{x_{i}^{n}\right\}}_{i=1}^{L_{n}}$, is converted into a set of continuous interpolation function collections $S^{n}\left(I\right)=\left\{S_{1}^{n}\left(I\right),S_{2}^{n}\left(I\right),\ldots ,S_{L_{n}-1}^{n}\left(I\right)\right\}$.

(2) $$\left\{\begin{array}{cc} S_{1}^{n}\left(I\right)=a_{1}I^{3}+b_{1}I^{2}+c_{1}I+d_{1} & \;I\in [1,2]\\ S_{2}^{n}\left(I\right)=a_{2}I^{3}+b_{2}I^{2}+c_{2}I+d_{2} & \;I\in [2,3]\\ \vdots & \\ S_{L_{n}-1}^{n}\left(I\right)=a_{L_{n}-1}I^{3}+b_{L_{n}-1}I^{2}+c_{L_{n}-1}I+d_{L_{n}-1} & \;I\in [L_{n}-1,L_{n}] \end{array}\right.$$

where *n* represents the group of effective respiratory cycles; $L_{n}$ denotes the length of the segment; and *a*, *b*, *c*, and *d* are the coefficients to be determined.

Subsequently, according to Equation (3), the data of the effective respiratory cycles ${\left\{x_{i}^{n}\right\}}_{i=1}^{L_{n}}$ are sequentially substituted into the interpolation functions, ensuring that the interpolation functions pass through each discrete data point.

(3) $$\left\{\begin{array}{cc} S_{k}^{n}\left(k\right)=x_{k}^{n} & \;k=1,2,3,\ldots ,L_{n}-1\\ S_{k}^{n}\left(k+1\right)=x_{k+1}^{n} & \;k=1,2,3,\ldots ,L_{n}-1 \end{array}\right.$$

Simultaneously, according to Equation (4), the first and second derivatives of adjacent interpolation functions at the same endpoints are made equal, ensuring that the adjacent interpolation functions have the same slope and curvature at the endpoints, resulting in a smoother transition.

(4) $$\left\{\begin{array}{cc} S_{k}^{n}{\left(k+1\right)}^{\prime }=S_{k+1}^{n}{\left(k+1\right)}^{\prime } & \;k=1,2,3,\ldots ,L_{n}-2\\ S_{k}^{n}{\left(k+1\right)}^{\prime \prime }=S_{k+1}^{n}{\left(k+1\right)}^{\prime \prime } & \;k=1,2,3,\ldots ,L_{n}-2 \end{array}\right.$$

Additionally, certain boundary conditions need to be applied at the start and end points of the interpolation functions [57]. According to Equation (5), the natural boundary [58] conditions are used in this research to set the second derivatives of the interpolation functions to zero at both the start and end points.

(5) $$\left\{\begin{array}{c} S_{1}^{n}{\left(1\right)}^{\prime \prime }=0\\ S_{L_{n}-1}^{n}{\left(L_{n}\right)}^{\prime \prime }=0 \end{array}\right.$$

By applying the above constraints, the undetermined coefficients can be solved, allowing the set of interpolation functions $S^{n}\left(I\right)$ to construct a globally smooth interpolation curve. This curve not only passes through all the data points but also avoids abrupt changes in slope and curvature at each data point, making it appear more continuous and smooth, both visually and functionally.

Finally, to obtain effective respiratory cycles of consistent length, the interpolation curve is resampled to generate fixed-length segments containing 2000 data points. Considering that the original sampling frequency was only 100 Hz, the resampled sequence with 2000 points corresponds to a significantly higher sampling frequency. This increased density not only satisfies the Nyquist sampling criterion for signal reconstruction but also smooths the effective respiratory cycle waveforms, thereby improving the accuracy of subsequent calculations. In addition, the 2000-point data length is compatible with the data format used in existing nasal resistance measurement systems, ensuring computational compatibility. According to Equation (6), the sampling interval $\Delta T^{n}$ is calculated based on the original length $L_{n}$ of each effective respiratory cycle and the length of the resampled data. Subsequently, the sampling points ${\hat{I}}_{j}$ are calculated according to Equation (7) and resampling is performed. In this research, each set of effective respiratory cycles was resampled into fixed-length segments containing 2000 data points, denoted as ${\left\{S^{n}\left({\hat{I}}_{j}\right)\right\}}_{j=1}^{2000}$.

(6) $$\Delta T^{n}=\frac{L_{n}}{2000}$$

(7) $$\left\{\begin{array}{c} {\hat{I}}_{1}=1\\ {\hat{I}}_{j+1}={\hat{I}}_{j}+\Delta T^{n}\;j=1,2,3,\ldots ,1999 \end{array}\right.$$

After processing, the effective respiratory cycles have the same length, enabling the rapid calculation of the average data across multiple tests. This method reduces errors from individual tests, making the measurement result of the 4-phase rhinomanometry more objective.

#### 2.2.5. Nasal Resistance Calculation Methods

The effective respiratory cycles and average data obtained from the preprocessing method are used to calculate various nasal resistance indicators. In the system proposed in this research, the classic rhinomanometry method, the 4-phase rhinomanometry method, and the Broms resistance measurement method can be used for nasal resistance calculation. This provides a quantitative basis for assessing nasal obstructions.

**(1)**  **Classic Rhinomanometry Method**

The mean resistance when breathing reaches the reference pressure can be directly calculated from effective respiratory cycles [36,52]. However, since the cycles consist of discrete data points, it is not always possible to ensure the presence of precise data points at the reference pressure within each effective respiratory cycle. Therefore, two data points near the reference pressure are selected, and the equation expression of their connecting line is derived. This allows the flow value at the reference pressure to be predicted. In this research, the mean resistance during inspiratory and mean resistance during expiratory were calculated separately. The mean resistance *R* at the reference pressure $P_{ref}$ is then determined using the specified Equation (8).

(8) $$R=\frac{P_{ref}}{V_{pred}}$$

where $P_{ref}$ is the reference pressure, typically set at 150 Pa or −150 Pa [31,37], and $V_{pred}$ is the predicted flow value at the reference pressure.

**(2)**  **4-phase Rhinomanometry Method**

**Figure 7:**
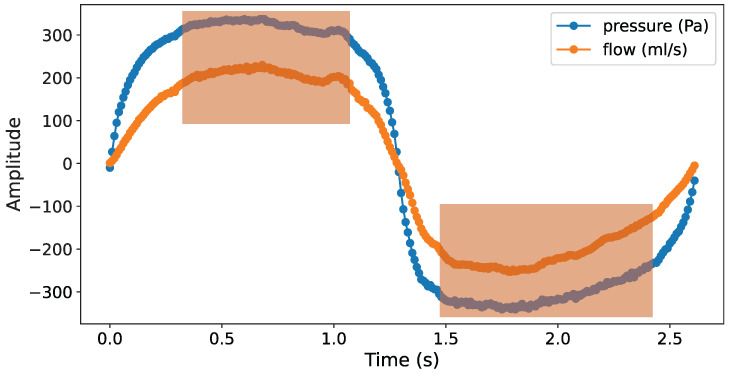
Stabilization curve of an effective respiratory cycle.

Vertex Resistance (VR) represents the nasal resistance when breathing reaches a steady state. This state is characterized by a linear relationship between pressure and flow signal as they become parallel on the curve. In Figure 7, during a set of effective respiratory cycles, there is one part of the stabilization curve in both the inspiratory and expiratory phases. Within the orange-shaded area in the figure, the flow reaches its peak, and the relationship between pressure and flow signal approximates a linear correlation. Compared to the non-steady state outside this area, the curve variation in the steady state is significantly smaller.

According to Equation (9), engineering practices typically use the resistance measured at the maximum flow rate [32]. In this research, the vertex resistance during inspiratory and vertex resistance during expiratory were calculated separately.

(9) $$R=\frac{P_{vertex}}{V_{vertex}}$$

where $V_{vertex}$ represents the maximum flow rate and $P_{vertex}$ denotes the pressure at which the maximum flow rate is achieved.

The term “Effective Resistance” was introduced into clinical rhinomanometry by Vogt and Hoffrichter in 1993 [32]. “Effective” values are calculated in electrical engineering using the equations for calculating energy in alternating current. An effective value is the integral of measured values over the time interval of interest:

(10) $$W_{eff}=\sqrt{\frac{1}{T}\int_{0}^{T}\omega^{2}dt}$$

According to Equation (10), the effective pressure $P_{eff}$ and the effective flow $V_{eff}$ can be calculated. By dividing these effective values according to Equation (11), the effective resistance is obtained. In this research, the effective resistance during inspiratory and effective resistance during expiratory were calculated separately.

(11) $$R=\frac{P_{eff}}{V_{eff}}$$

**(3)**  **Broms Resistance Measurement Method**

**Figure 8:**
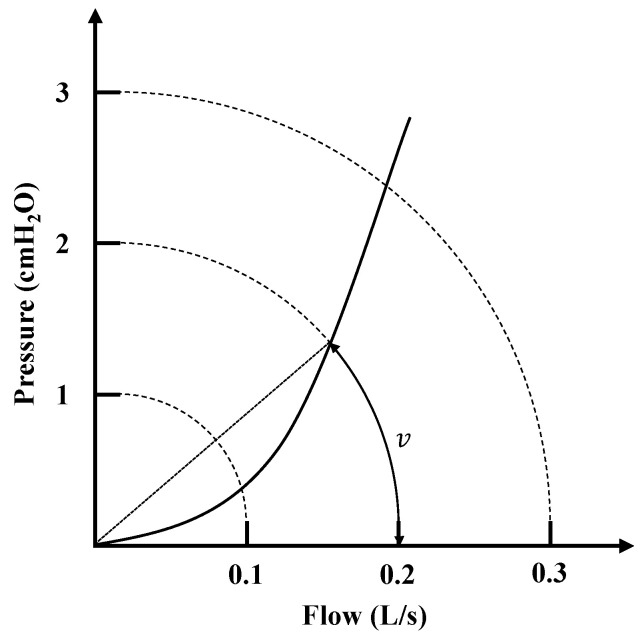
Intersection of curves in Broms resistance measurement method.

The Broms resistance measurement method is commonly used for pediatric patients, enhancing the general applicability of nasal resistance measurement. According to the experiment by Vogt et al. [53], Broms resistance is defined as the resistance at the intersection of the pressure–flow curve with a unit circle of radius 2. As shown in Figure 8, the calculation of the Broms resistance requires first determining the angle formed between the pressure–flow curve and the circle. This angle is then converted into a resistance value.

Since this resistance calculation first involves determining the angle between pressure and flow, the pressure–flow curve can be converted into polar coordinates. According to Equation (12), the angle values corresponding to a specific radius can be directly predicted.

(12) $$v_{pred}=v_{0}+cr$$

where $v_{pred}$ is the predicted angle value, *r* is the reference radius, typically set to 2 [4,53], and $v_{0}$ and *c* are learnable parameters.

During the resistance calculations, the data points near the reference radius are first selected from effective respiratory cycles as the training dataset. Then, the least squares method is used to estimate the learnable parameters, and the parameter combination that minimizes error is identified. This allows the angle $v_{pred}$ of the pressure–flow curve at a reference radius of 2 to be predicted. Finally, the tangent value of the angle $v_{pred}$ is calculated to represent the Broms resistance value.

## 3. Results

### 3.1. Results and Analysis of the Threshold Search

**Figure 9:**
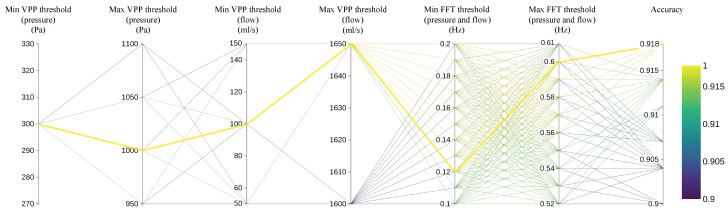
Classification accuracy of respiratory cycles with different threshold combinations.

To provide a more intuitive demonstration of the impact of different threshold values on the final classification accuracy of respiratory cycles and to identify the optimal threshold combination, the process of selecting threshold values was illustrated using a parallel coordinate plot. Since the threshold selection involves a multidimensional iterative search, the parallel coordinate plot provides an effective way to visualize both input and output dimensions simultaneously. The first six axes correspond to the input thresholds, while the last axis indicates the classification accuracy. The process is based on 280 respiratory cycles, with each cycle evaluated by a physician to determine whether or not it is considered an effective respiratory cycle. As shown in Figure 9, the classification accuracy corresponding to various values of the six thresholds—Min VPP threshold (pressure), Max VPP threshold (pressure), Min VPP threshold (flow), Max VPP threshold (flow), Min FFT threshold (pressure and flow), and Max FFT threshold (pressure and flow)—is displayed. Given the vast number of possible threshold combinations, only the top 1000 threshold sets with the highest accuracy are represented in the figure. The yellow lines represent the threshold sets that achieve the highest accuracy, reaching up to 91.8%. By analyzing all the yellow lines, it can be concluded that the optimal value for Min VPP threshold (pressure) is 300 Pa, for Max VPP threshold (pressure) is 1000 Pa, for Min VPP threshold (flow) is 100 mL/s, and for Max VPP threshold (flow) is 1650 mL/s. Additionally, the optimal classification accuracy is achieved when the Min FFT threshold (for both pressure and flow) is set to 0.12 Hz and the Max FFT threshold (for both pressure and flow) is set to 0.6 Hz, thereby maximizing the frequency range of effective respiratory cycles.

### 3.2. Results and Analysis of the Time–Frequency-Domain Analysis Algorithm

**Figure 10:**
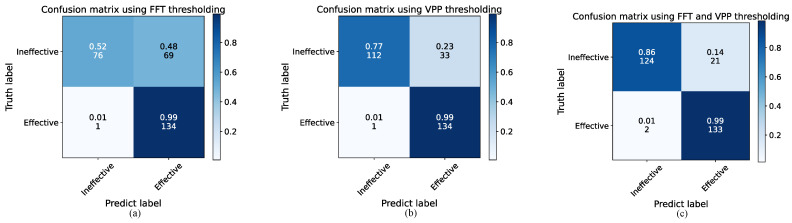
Confusion matrices for different preprocessing methods: (**a**) Using FFT thresholding, (**b**) Using VPP thresholding, and (**c**) Using both FFT and VPP thresholding.

To validate the accuracy of the proposed preprocessing method for distinguishing between effective and ineffective respiratory cycles, an ablation experiment was conducted using 280 sets of respiratory cycle data, with each set labeled by a physician as either an effective or ineffective respiratory cycle. The recognition performance was compared and analyzed across different preprocessing methods. Specifically, the recall and precision rate of three preprocessing methods were evaluated: using the FFT threshold alone, using the VPP threshold alone, and using a combination of FFT and VPP thresholds. The results were presented by confusion matrices, as shown in Figure 10a–c. The vertical axis represents the true labels, while the horizontal axis represents the predicted labels. The color of each cell varies from light to dark, indicating an increasing number of samples, and each cell displays both the sample count and the percentage.

Based on Figure 10a, it is clear that the preprocessing method using only the FFT threshold works well in recognizing effective respiratory cycles, with a recall rate of 99%. This means that the method is able to retain most of the effective respiratory cycles. However, its precision in recognizing effective respiratory cycles is relatively low at 66%, indicating that it struggles to filter out ineffective respiratory cycles. This difficulty arises from the close frequency range between some ineffective respiratory cycles and effective respiratory cycles, making accurate identification challenging when relying solely on the FFT threshold. Figure 10b shows that the preprocessing method using only the VPP threshold also achieves good performance in recognizing effective respiratory cycles. Additionally, this method improves the exclusion of ineffective respiratory cycles, maintaining a recall rate of 99% for effective respiratory cycles while increasing the precision to 80%. Finally, the performance of the preprocessing method combining both FFT and VPP thresholds was analyzed, as shown in Figure 10c. This combined approach ensures a high recall rate of 99% for effective respiratory cycles while further enhancing the precision to 86%. A 20% improvement in precision is represented compared to the use of the FFT threshold alone. These results indicate that the preprocessing method’s ability to distinguish between effective and ineffective respiratory cycles is significantly improved by using both threshold conditions. This method enhances precision while maintaining high recall rate, effectively retaining effective respiratory cycles and eliminating ineffective ones.

In addition to the overall assessment of the preprocessing method’s effectiveness in identifying effective and ineffective respiratory cycles, this research also evaluates the accuracy of recognizing ineffective respiratory cycles under different interference scenarios to verify the reliability of the proposed method. The experiment analyzed the performance under three common interference scenarios defined in Section 2.2.1: saturated or weak breaths, breaths when not worn properly, and multiple breaths.

**Figure 11:**
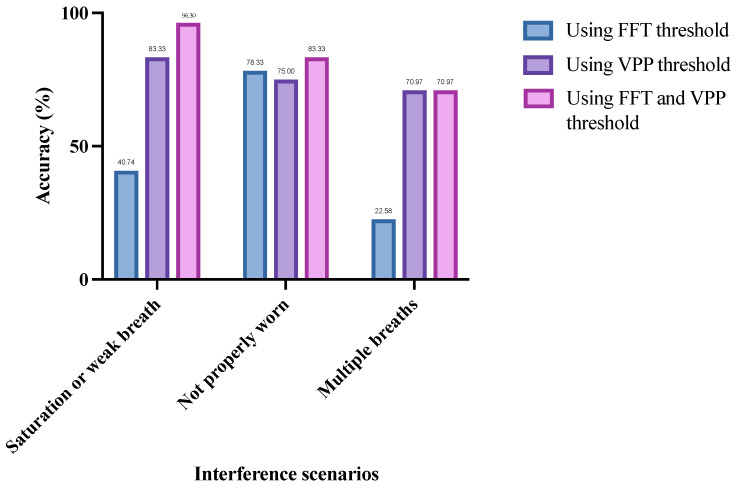
Recognition accuracy of ineffective respiratory cycles by different preprocessing methods in three interference scenarios.

As shown in Figure 11, for the saturated or weak breaths interference scenario, the preprocessing method using the FFT threshold alone achieved an accuracy of only 40.74% for recognizing ineffective respiratory cycles. When using the VPP threshold alone, the accuracy increased to 83.33%. When both thresholds were combined, the accuracy further improved to 96.30%, representing a 55.56% increase compared to using the FFT threshold alone and a 12.97% increase compared to using the VPP threshold alone. This indicates that the VPP threshold condition is more critical in this interference scenario. The reason is that most saturated or weak breaths are generated by the testee’s normal breathing, but the intensity of the breathing either exceeds the measurement range of the equipment or does not meet the required strength for computation. Therefore, in this scenario, the frequency-domain results of most respiratory cycles conform to the standards of effective respiratory cycles, making them more sensitive to the VPP threshold.

In contrast to the saturated or weak breaths interference scenario, in the scenario when the breathing device is not worn properly the preprocessing method using the FFT threshold alone achieves a 3.33% higher accuracy compared to using the VPP threshold alone. When both thresholds are combined, the accuracy can be further increased to 83.33%. This indicates that, in this interference scenario, the FFT threshold condition is more critical for accurately identifying ineffective respiratory cycles. This is because, when the breathing device is not worn properly, the signals collected by the lower computer may not originate from the testee’s breathing but rather from air fluctuations caused during the wearing process. These signals are highly random and are more likely to fall outside the FFT threshold range. Therefore, the FFT threshold is more sensitive in this scenario.

Additionally, during multiple breaths interference, the preprocessing method using the VPP threshold alone achieves an accuracy of 70.97% for recognizing ineffective respiratory cycles, which is a 48.39% improvement compared to using the FFT threshold alone. The combined use of both thresholds also reaches an accuracy of 70.97%. This is likely because multiple breaths interference often occurs during the inspiratory or expiratory phases of normal breathing, primarily affecting either the inspiratory or expiratory signals without significantly altering the overall breathing pattern. As a result, the frequency distribution of the entire respiratory cycle may be similar to that of the effective respiratory cycle, making it less sensitive to the FFT threshold.

In summary, as shown in Figure 11, the saturated or weak breaths interference scenario and the multiple breaths interference scenario are more sensitive to the VPP threshold, while the breathing device not worn properly scenario is more sensitive to the FFT threshold. Additionally, regardless of the interference scenario, the preprocessing method that combines both FFT and VPP thresholds consistently achieves the highest recognition accuracy. This indicates that the proposed preprocessing method effectively leverages the advantages of both FFT and VPP thresholds, making it suitable for various interference scenarios.

### 3.3. Results and Analysis of Nasal Resistance Calculation Methods

#### 3.3.1. Stability Analysis of Measurement Results

In clinical practice, nasal resistance in patients is commonly quantified using indicators such as vertex resistance, effective resistance, mean resistance, and Broms resistance. The results for these four types of resistance are derived from the same respiratory cycles during calculation. To further analyze the impact of preprocessing methods on the calculation results of these four resistances, an experiment was conducted involving 45 groups of respiratory data, each containing at least four effective respiratory cycles. The four types of resistance were calculated and recorded for each respiratory cycle under two preprocessing methods: without respiratory cycle filtering and with respiratory cycle filtering. Subsequently, the standard deviation of the resistance results within each group was calculated and recorded. The distribution of the within-group standard deviation of resistance for the 45 groups of respiratory data was then statistically analyzed and presented using boxplots. In each boxplot, the median is indicated by a solid line within the box, and the first and third quartiles of the distribution are at the edges of the box. The distance between the first and third quartiles is referred to as the Interquartile Range (IQR) and is used to represent the concentration of the distribution.

**Figure 12:**
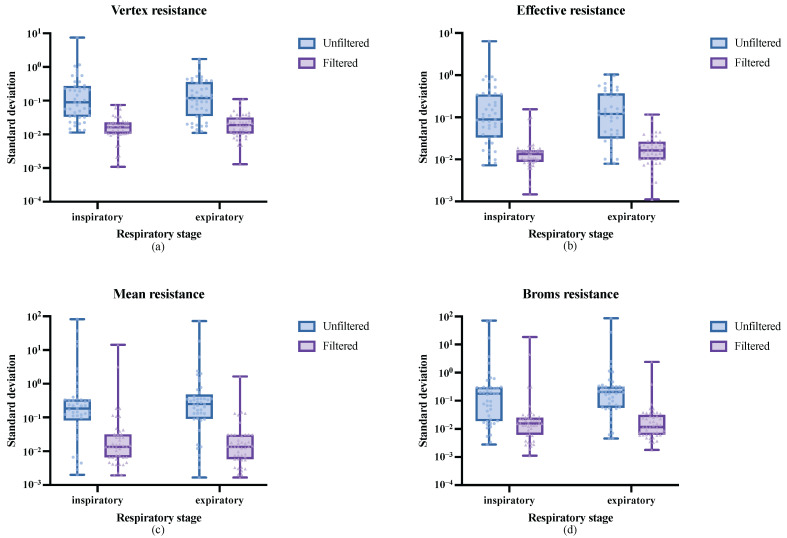
Within-group standard deviation distribution of the four types of nasal resistance measurement results under different preprocessing methods: (**a**) Vertex resistance, (**b**) Effective resistance, (**c**) Mean resistance, and (**d**) Broms resistance.

As shown in Figure 12a, the standard deviation distribution of vertex resistance for all datasets is illustrated. The experiment separately analyzed the inspiratory and expiratory phases, with blue representing the standard deviation distribution without respiratory cycle filtering and purple representing the standard deviation distribution after respiratory cycle filtering. It can be observed that, without filtering, the standard deviation of vertex resistance is relatively large and widely distributed. The IQR for the inspiratory and expiratory phases are 0.2416 and 0.3223, respectively. The medians of the standard deviation of vertex resistance for the inspiratory and expiratory phases are 0.0896 and 0.1196, respectively. However, after respiratory cycle filtering, the standard deviations of vertex resistance in both the inspiratory and expiratory phases significantly decreased, with a narrower distribution range. The IQR for the inspiratory phase decreased by 20 times, reducing to 0.0122, and the IQR for the expiratory phase decreased by 15 times, reducing to 0.0209. Additionally, the minimum standard deviations for both phases were significantly reduced to 0.001. The medians of the standard deviations for the inspiratory and expiratory phases decreased by five times and six times, respectively, reducing to 0.0161 and 0.0188. As shown in Figure 12b, the standard deviation distribution of effective resistance is presented. The figure indicates that, after respiratory cycle filtering, the standard deviations of the calculated results for both the inspiratory and expiratory phases significantly decreased, with the medians reducing seven times, reducing to 0.0133 and 0.0163, respectively. Furthermore, as depicted in Figure 12c,d, for mean resistance and Broms resistance, respectively, the median standard deviations after respiratory cycle filtering decreased by 11 to 18 times.

Overall, the preprocessing method proposed in this research effectively filters respiratory cycles by automatically removing ineffective respiratory cycles affected by interference, leaving only effective respiratory cycles as high-quality data sources for calculating the four types of nasal resistance. The removal of ineffective respiratory cycles eliminates abnormal respiratory data caused by interference during measurement, thus reducing the impact of the results of various nasal resistance indicators.

#### 3.3.2. Example Analysis of Measurement Results

The measurement results after respiratory cycle filtering can more objectively describe the true condition of the testee. As shown in Table 1, this research provides a representative example of measurement results for a patient with nasal obstruction, which demonstrates the importance of respiratory cycle filtering. In medical diagnosis, it is considered that a resistance value below 0.75 in the 4-phase rhinomanometry indicates normal nasal function, while a value above 0.75 is classified into different grades of nasal obstruction [59,60].

As shown in Table 1, different preprocessing methods can alter the final measurement results of nasal resistance. When respiratory cycle filtering is applied, the resistance values during the inspiratory phase are consistently lower than those during the expiratory phase across four types of resistance. Moreover, for vertex resistance and effective resistance, the resistance values during the expiratory phase exceed 0.75. This value is above the normal reference range, indicating nasal obstruction. These results align with the actual condition of the testee. However, without respiratory cycle filtering, the resistance values for vertex resistance and effective resistance are relatively close during the inspiratory and expiratory phases. Additionally, for mean resistance and Broms resistance, the resistance during the inspiratory phase is significantly higher than during the expiratory phase. Furthermore, the vertex resistance and effective resistance values are both below 0.75, suggesting normal nasal function, which contradicts the true condition of nasal obstruction in the testee.

The results indicate that, without respiratory cycle filtering, the final measurement results may significantly deviate from the actual condition due to interference from ineffective respiratory cycles. This interference can particularly lead to misjudgment of the severity of nasal obstruction. In contrast, the preprocessing method proposed in this research effectively filters out ineffective respiratory cycles, ensuring the accuracy of subsequent nasal resistance calculations and providing a more objective reflection of the patient’s actual respiratory state.

## 4. Discussion

Currently, the reproducibility and repeatability of classic rhinomanometry methods (such as the anterior active rhinomanometry method) and the 4-phase rhinomanometry method have become a major focus of research. Several studies conducted stability analyses based on anterior active rhinomanometry measurement results, exploring the repeatability of nasal resistance measurements under different statistical methods [34]. In addition, some studies examined the impact of factors such as gender, age, and height on nasal resistance measurements, as well as the effect of different test intervals on measurement repeatability [61]. Furthermore, certain studies compared nasal resistance measurement methods based on pressure and flow with those from acoustic rhinometry and CFD methods, analyzing the reliability and consistency of different approaches [23,24]. However, these studies primarily focus on the application analysis of various nasal resistance measurement results or the impact of different subject conditions on the measurement outcomes, with relatively few studies addressing the optimization of nasal resistance measurement methods themselves.

In contrast to previous research, this study focuses on optimizing the nasal resistance measurement method itself, aiming to improve the stability and anti-interference capabilities of the measurement system. In the experiments, effective and ineffective respiratory cycles were efficiently identified using a time–frequency-domain analysis algorithm by appropriately setting the VPP and FFT thresholds. The experimental results show that using high-quality effective respiratory cycles as data sources for calculating resistance indicators can significantly improves the stability of the measurement results.

Regarding the analysis of respiratory data, a method based on statistical Standard Error of Mean (SEM) was proposed by Carney et al. to filter respiratory data, ensuring the stability and repeatability of the measurement results by discarding resistance values exceeding twice the SEM [62]. However, this method primarily relied on mean and variance calculations, and it may take some time for the data to stabilize. In contrast, we delve into the characteristics of disturbed, ineffective respiratory cycles during nasal resistance measurement and propose a filtering method based on time–frequency-domain analysis. Our method can directly filter raw collected data, fundamentally reducing interference. Unlike traditional methods that rely on mean and variance calculations, the time–frequency-domain analysis algorithm proposed in this study offers significant advantages. It enables real-time data processing without the need for large sample accumulations, allowing for immediate analysis and filtering of data during the measurement process. Therefore, our approach can improve measurement efficiency while maintaining the stability of the results.

In addition to single-source interference, the nasal resistance measurement system may encounter composite or overlapping disturbances during actual data acquisition. When multiple interference sources act simultaneously, the system captures the most prominent interference feature and classifies the respiratory cycle according to the most probable disturbance pattern. Although this classification approach may have limitations in precisely distinguishing all overlapping interference types, the affected respiratory cycle is still labeled as ineffective and excluded from resistance calculations. This design ensures that such complex interference does not compromise the reliability of the final nasal resistance measurement. Meanwhile, boundary disturbances—which refer to signal cycles that fall near threshold limits—are also handled through the optimized threshold selection process. By identifying the most appropriate combination of FFT and VPP thresholds, these borderline cases are reliably excluded as ineffective respiratory cycles. This approach not only improves the robustness of the data filtering mechanism but also enhances the accuracy and consistency of the final nasal resistance results.

In terms of the nasal resistance measurement system, the system designed in this study enhances the data processing capabilities of the upper computer analysis software. Compared to conventional measurement systems, this system features a higher level of automation and stronger anti-interference capabilities. While meeting the requirements for calculating various nasal resistance indicators, it significantly improves system usability and stability. Future research could integrate auxiliary diagnostic models for diseases caused by abnormal nasal resistance into the measurement system, further enhancing the system’s level of intelligence.

## 5. Conclusions

In this research, a multi-sensor fusion system was designed for the nasal resistance measurement based on pressure and flow, comprising the lower computer of a nasal pressure–flow signal acquisition device and the upper computer of nasal pressure–flow signal analysis software. The raw pressure–flow signals generated during breathing were acquired by the lower computer. The upper computer software utilized the nasal pressure–flow signal preprocessing method to synchronize the collection and segmentation of respiratory cycles from the raw pressure–flow signals in real-time. The characteristics of undisturbed effective respiratory cycles and disturbed ineffective respiratory cycles were analyzed. Additionally, the impact of ineffective respiratory cycles on nasal resistance measurement results was assessed. Through multiple experiments, the time-domain and frequency-domain features of the respiratory cycles were analyzed, and the proposed time–frequency-domain analysis algorithm was employed to filter out effective respiratory cycles, thereby reducing the influence of ineffective ones. The results indicate that the nasal resistance measurement system developed in this research demonstrates strong anti-interference capabilities, significantly enhances the automation of the measurement process and the stability of the measurement results, and offers more objective results for assessing nasal resistance. Moreover, the upper computer software provides an intuitive display of the results from all nasal resistance calculation methods and the pressure–flow curves.

The system proposed in this research is suitable for nasal resistance measurement scenarios using the classic rhinomanometry method, the 4-phase rhinomanometry method, and the Broms resistance measurement method. In practical applications, this system can automatically process and analyze the collected data, eliminating ineffective respiratory cycles to improve the calculation of nasal resistance indicators. However, there are still some issues with the current multi-sensor fusion nasal resistance measurement system. Building on the foundation of this research, the stability and measurement accuracy of the system can be further improved by several methods. Firstly, more interference scenarios need to be considered to expand the types of ineffective respiratory cycles. Additionally, the time–frequency domain analysis algorithm proposed in this research only utilizes the FFT threshold and VPP threshold to identify effective respiratory cycles, which may be less effective in more complex interference scenarios. These challenges need to be addressed to further improve the nasal resistance measurement system.

## Figures and Tables

**Table 1:** Comparison of nasal resistance measurement results under different preprocessing methods.

Preprocessing Methods	Vertex Resistance		Effective Resistance		Mean Resistance		Broms Resistance	
	Pa/cm^3^/s		Pa/cm^3^/s		Pa/cm^3^/s		Pa/cm^3^/s	
	Ins *	Exp *	Ins *	Exp *	Ins *	Exp *	Ins *	Exp *
Unfiltered	0.600	0.593	0.601	0.599	1.078	0.395	0.735	0.470
Filtered	0.729	0.791	0.701	0.763	0.481	0.548	0.387	0.475

* Ins: Inspiratory; Exp: Expiratory.

**Figure A1:**
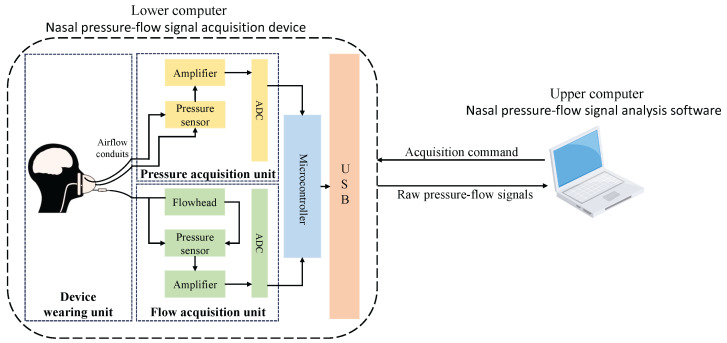
Lower computer hardware architecture diagram.

**Figure A2:**
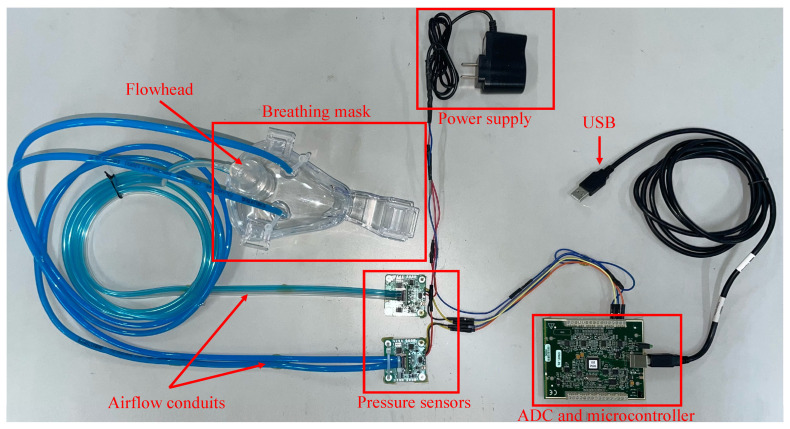
Lower computer hardware physical connection diagram.

**Figure A3:**
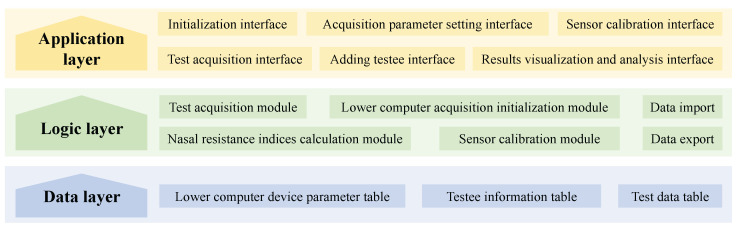
Upper computer software architecture diagram.

**Figure A4:**
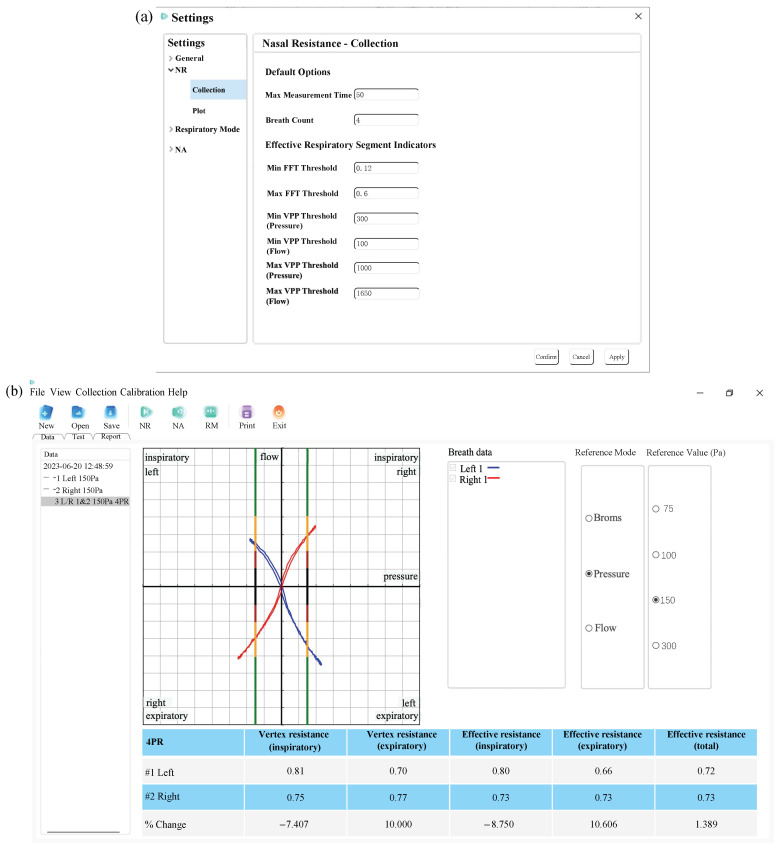
(**a**) Acquisition parameters setting interface. (**b**) Result visualizing interface.

## Data Availability

The data that support the findings of this study are available from the corresponding author upon reasonable request.

## Appendix A

### Appendix A.1. The Lower Computer of Nasal Pressure–Flow Signal Acquisition Device

The structure of the lower computer nasal pressure–flow signal acquisition device is shown in Figure A1. Its basic structure includes the following components: a device-wearing unit, which includes a breathing mask, medical nasal plugs, and airflow conduits; a pressure acquisition unit composed of a pressure sensor, signal amplifier, and ADC; a flow acquisition unit composed of a flowhead, pressure sensor, signal amplifier, and ADC; a microcontroller; a USB communication interface. The pressure sensor used in the system is the model 163PC01D36-PC from First Sensor. This high-precision differential pressure sensor supports a measurement range of ±1.25 kPa and maintains an accuracy of ±2% Full Scale (FS), making it well-suited for low-pressure respiratory signal monitoring. These five components respectively implement the functions of wearing the breathing device, acquiring pressure signals, acquiring flow signals, data processing, and data transmission. The physical picture of the lower computer nasal pressure–flow signal acquisition device is shown in Figure A2.

The workflow of the lower computer nasal pressure–flow signal acquisition device is as follows: The upper computer software sends acquisition commands to the lower computer through the USB interface. Upon receiving the command, the microcontroller initializes the ADCs of the pressure acquisition unit and the flow acquisition unit, both set to single-ended sampling mode, preparing for data collection. The pressure and flow generated by the testee breathing are transmitted through the breathing mask and airflow conduits to the two acquisition units. The pressure changes are directly acquired by the pressure sensor and quantified by the ADC. The flow changes are first converted to pressure changes by the flowhead, then acquired by the pressure sensor and quantified by the ADC. Finally, the raw pressure–flow signals are processed by the microcontroller and transmitted back to the upper computer through the USB interface for further processing.

### Appendix A.2. The Upper Computer of Nasal Pressure–Flow Signal Analysis Software

The upper computer software architecture is shown in Figure A3. The software is primarily composed of three parts, arranged from bottom to top: the data layer, the logic layer, and the application layer.

In the data layer, essential data required for the software’s operation are stored, which includes the lower computer device parameter table, the testee information table, and the test data table. These tables respectively store the necessary configuration data for communication with the lower computer, the basic personal information of the testee, and detailed test data along with result analyses. When the test acquisition function is executed, the test data can be stored in the corresponding data table based on the testee information, facilitating future use for the same testee. This layer serves as the foundation of the software, providing structured data storage space, enabling convenient management of test data, and enhancing the software’s operational efficiency.

In the logic layer, various functional modules are implemented, such as the lower computer acquisition initialization module, the sensor calibration module, the test acquisition module, and the nasal resistance indicators calculation module. This layer establishes the communication process with the lower computer and facilitates data transmission. It utilizes detailed test data from the data layer to implement nasal pressure–flow signal processing methods for calculating various nasal resistance indicators. Additionally, the logic layer enables data import and export. This layer provides a rich and efficient means for implementing the software’s various functions.

In the application layer, the primary focus is on the user interaction interface, which includes the initialization interface, adding testee interface, sensor calibration interface, test acquisition interface, acquisition parameter setting interface, and results visualization and analysis interface. The application layer displays data and information to the user through controls such as forms, images, and tables. At the same time, It also receives user commands and information through controls such as buttons and text input boxes. The application layer interacts directly with the user, serving as a convenient interaction medium between the user and the measurement system.

### Appendix A.3. Key Functional Interface of Upper Computer Software Platform

The main functional interfaces of the upper computer software are shown in Figure A4. The figure illustrates the acquisition parameters setting interface and the result visualizing interface. As shown in Figure A4a, the acquisition parameter setting interface allows users to control the performance of the nasal pressure–flow signal preprocessing method by setting parameters such as FFT threshold, VPP threshold, and maximum measurement time. As depicted in Figure A4b, the result visualization interface displays the pressure–flow curves for the left and right nasal cavities using blue and red lines, respectively, according to medical standards. Additionally, the operator can use different functional buttons to perform calculations for the classic rhinomanometry, the 4-phase rhinomanometry, and the Broms resistance. The results of each nasal resistance calculation are presented in tabular form.
